# Marmoset superior colliculus: neuronal expression of somatostatin but not vasoactive intestinal peptide or neuropeptide Y

**DOI:** 10.3389/fnana.2025.1731419

**Published:** 2025-12-11

**Authors:** Melissa H. Y. Chong, Emmanuel K. L. Cho, Marcello G. P. Rosa, Nafiseh Atapour

**Affiliations:** Department of Physiology and Neuroscience Program, Biomedicine Discovery Institute, Monash University, Melbourne, VIC, Australia

**Keywords:** superior colliculus, somatostatin, neuropeptide Y (NPY), vasoactive intestinal peptide (VIP), primate

## Abstract

The superior colliculus (SC) is a layered midbrain structure that plays a crucial role in integrating sensory information toward functions such as directing eye and head movements. Despite significant literature on its anatomical structure and connections, there are still important gaps in our knowledge of the diversity of cell types, particularly in primates. Here, using immunostaining, we examined the expression of three different neuropeptides [somatostatin (SST), vasoactive intestinal peptide (VIP), and neuropeptide Y (NPY)] in the SC of adult marmoset monkeys (*Callithrix jacchus*). We found neurons expressing SST (SST-positive, SST+) across all cellular layers of the SC, which corresponded to approximately 3-5% of the total neuronal population in this structure. SST+ neuronal density as estimated by stereological sampling methods was about 3,140/mm^3^ in the top layer, stratum griseum superficiale (SGS) and decreased across the dorsoventral axis, roughly in line with the overall neuronal density estimated from NeuN stained nuclei. Co-staining of SST with gamma-aminobutyric acid (GABA), confirmed the inhibitory nature of these cells. However, we found no evidence of VIP- or NPY-positive neurons in the marmoset SC, despite the presence of clearly stained neurons in other structures, in the same sections. Our data adds to the understanding of neuronal diversity of SC in primates and provides quantitative estimates of SST+ neurons in this structure that is essential for better understanding of its function and phylogeny.

## Introduction

The superior colliculus (SC), a midbrain structure made up of seven horizontal layers, integrates diverse sensory inputs to generate motor commands (Allen et al., 2021; Corneil and Munoz, 2014; Isa et al., 2021; White and Munoz, 2012), while also contributing to higher-order processes such as visual spatial attention and cognition (Basso et al., 2021; Krauzlis et al., 2013). The superficial layers of the SC, including the stratum griseum superficiale (SGS) and stratum opticum (SO), are visual layers (May, 2006; Liu et al., 2022; Rosa and Schmid, 1994) which receive direct inputs from the retina and visual cortex and send outputs to multiple subcortical structures (Basso et al., 2021; Cang and Feldheim, 2013; Huberman et al., 2009; Wang and Burkhalter, 2013). Neurons in the intermediate (stratum griseum intermedium, SGI and stratum album intermedium, SAI) and deep layers (stratum griseum profundum, SGP and stratum album profundum, SAP) respond to stimulus modalities in addition to vision, and are involved in integrating multimodal sensory information, cognitive inputs, and motor signals (Basso et al., 2021; Corneil and Munoz, 2014; White and Munoz, 2012; Krauzlis et al., 2013).

Single-cell genomics have unraveled the diverse molecular cell types of SC (Tsai et al., 2022; Xie et al., 2021; Liu et al., 2023), including various excitatory and inhibitory neuronal types. Among them are neurons expressing calcium-binding proteins including calbindin (CB), calretinin (CR), and parvalbumin (PV), which we have previously described quantitatively in SC of marmoset monkeys (Chong et al., 2022). To further understand SC neurochemistry in this species of primate, here we examined the expression of three different neuropeptides, somatostatin (SST), vasoactive intestinal peptide (VIP) and neuropeptide Y (NPY).

Transcriptomic data have suggested of presence of SST, VIP and NPY neurons in the mouse SC (Liu et al., 2023; Chen et al., 2025; Byun et al., 2016). Yet, data in the marmoset (Shimogori et al., 2018; Kita et al., 2021) have indicated gene expression for SST but not VIP or NPY in adult animals. However, immunohistochemistry data for these neuropeptides, which are important for revealing protein content, remain scarce in the primate SC, in general. Presence of SST-positive (SST+) neurons in SC of rodents (Laemle et al., 1982; Harvey et al., 2001; Byun et al., 2016), cat (Spangler and Morley, 1987) and several other species (Mensah-Brown and Garey, 2006; De Souza et al., 2015) has been demonstrated. While VIP expression has also been observed in the SC of rodents (Dussaillant et al., 1992; Ogawa-Meguro et al., 1992; Okamoto et al., 1990), only VIP-positive (VIP+) fibers—rather than cell bodies—have been reported in the SC of cats (Borostyánkoi et al., 1999). There are more mixed results on the presence of NPY-positive (NPY+) neurons in SC. In contrast to observations of NPY+ neurons in the cat (Coveñas et al., 1990) and hamster (Morin and Blanchard, 1997) SC, Borostyánkoi et al. (1999) reported only NPY+ neuronal fibers in the SC of cats.

To fill the gap in literature, we aimed to address the expression of SST, VIP, and NPY in the SC of the marmoset monkey by immunostaining. Our quantitative analysis can be a useful resource to guide future studies of SC structure and function, and indicate phylogenetic differences in neuronal circuitry, despite the apparently conserved cytoarchitecture (Rosa and Schmid, 1994; Bourne and Rosa, 2003).

## Materials and methods

Materials were obtained from seven young adult marmosets aged between 29 and 38 m (Table 1). The experiments were conducted in accordance with the Australian Code of Practice for the Care and Use of Animals for Scientific Purposes. All procedures were approved by the Monash University Animal Ethics Experimentation Committee, which also monitored the health and wellbeing of the animals throughout the experiments. The animals had no veterinary record of serious or chronic health conditions.

**Table 1:** Details of subjects used for each immunostaining.

**Subject (sex)**	**Age at perfusion (months)**	**SST**	**VIP**	**NPY**	**SST & GABA**	**NeuN**
CJ216 (F)	38	-	-	-	-	✓
CJ226 (F)	29	✓	✓	-	-	-
CJ227 (M)	37	✓	✓	-	-	-
CJ240 (M)	36	✓	✓	✓	-	-
CJ243 (M)	33	-	-	✓	-	-
CJ249 (F)	35	-	-	✓	✓	-
CJ255 (F)	33	-	-	-	✓	-

SST, somatostatin; VIP, vasoactive intestinal peptide; NPY, neuropeptide Y; GABA, gama amino butyric acid; NeuN, neuronal nuclei; F, female; M, male.

Subjects were overdosed using sodium pentobarbitone (100 mg/kg, i.v.), and transcardial perfusion was performed using heparinised saline, followed by 4% paraformaldehyde (PFA) in 0.1 M phosphate buffer solution (PBS) (Atapour et al., 2017). The collected brains were postfixed in the same medium for 1 hr (for VIP and SST staining) or overnight (for NPY and NeuN staining), after which cryoprotection was performed by submerging the brains in PBS (for VIP and SST staining) or PFA (for NPY and NeuN staining) with increasing concentrations of sucrose (10%, 20%, and 30%), over several days at 4 °C. Using a cryostat, frozen 40 μm coronal sections were obtained.

### Immunostaining

The sections were incubated in blocking solution (SST & VIP: 10% normal horse serum, NPY: 10% normal goat serum; all solutions containing 0.3% Triton-X100 in 0.1 M PBS) for 1 hr at room temperature before undergoing primary antibody [Anti-mouse SST (H-11, Santa Cruz, RRID:AB_2271061, 1:500); Anti-mouse VIP (Ab30680, Abcam, (RRID:AB_778830, 1:500); Anti-rabbit NPY (N9528, Sigma-Aldrich, RRID:AB_260814, 1:15000); Anti Neuronal Nuclei (NeuN, MAB377 Clone A60; Millipore, RRID: AB_2298772. 1:800)] incubation at 4 °C for 42–46 h. A biotinylated IgG secondary antibody [SST, VIP & NeuN: horse anti-mouse (PK-6102, VECTASTAIN^®^ Elite^®^ ABC-HRP Kit,1:200, RRID: AB_2336821); NPY: goat anti-rabbit (PK-6101, VECTASTAIN^®^ Elite^®^ ABC-HRP Kit, 1:200, RRID: AB_2336820)] incubation was then conducted for 30 min, followed by treatment with Avidin-Biotin Complex reagent. 3, 3′-diaminobenzidine (DAB) substrate working solution (DAB kit SK-4100, RRID: AB_2336382) was then applied. Given that different isoforms of SST might be present in variable cells, we selected an SST antibody that detects the 14–amino acid active peptide and any larger precursors or peptides containing it, such as SST-28 (Bakhit et al., 1984).

For co-immunofluorescence staining of GABA and SST, we used sections with only 1 h post-fixation and processed them for 42-46 h incubation at 4 °C with primary antibodies [SST (H-11), 1:100 and anti-rabbit GABA (A2052, Sigma-Aldrich, RRID: AB_477652)] before application of the secondary antibodies [1:600; Alexa Fluor^®^ 488 (ab150109, RRID: AB_2571721) and Alexa Fluor^®^ 594 (ab150064, RRID: AB_2734146)] for 60 min at room temperature.

### Gallyas silver stain for myelin

After cryosectioning, tissue sections were post-fixed in 4% buffered formalin for minimum 2 weeks and then mounted on double gel-subbed slides out of warm buffered 0.3% gelatine solution. They were air dried for 5-7 days before staining. Details of solutions and steps for staining were as described previously (Gallyas, 1979, modified by Worthy et al., 2017).

### Cresyl violet stain for Nissl bodies

After cryosectioning, tissue sections were post-fixed in 4% buffered formalin for 1-2 weeks, then mounted on gel-subbed slides out of warm buffered 0.5% gelatine solution. They were air dried for 5-7 days before defatting overnight in 50:50 chloroform and 100% ethanol. Sections were rinsed in 100% ethanol for 5 min, then placed into xylene for 3 h. Sections were then taken through dehydration and rehydration steps (Powers and Clark, 1955; modified by Worthy and Burman, 2017) into distilled water. From there, sections were placed in filtered cresyl violet at 38-40 °C for 8-10 mins. Then slides were removed and went back through distilled water and increasing gradient of ethanol, followed by differentiation in acidified 70% alcohol to remove background and make both SC layers and cell nuclei more distinguishable.

### Stereological cell sampling and statistics

**Figure 1:**
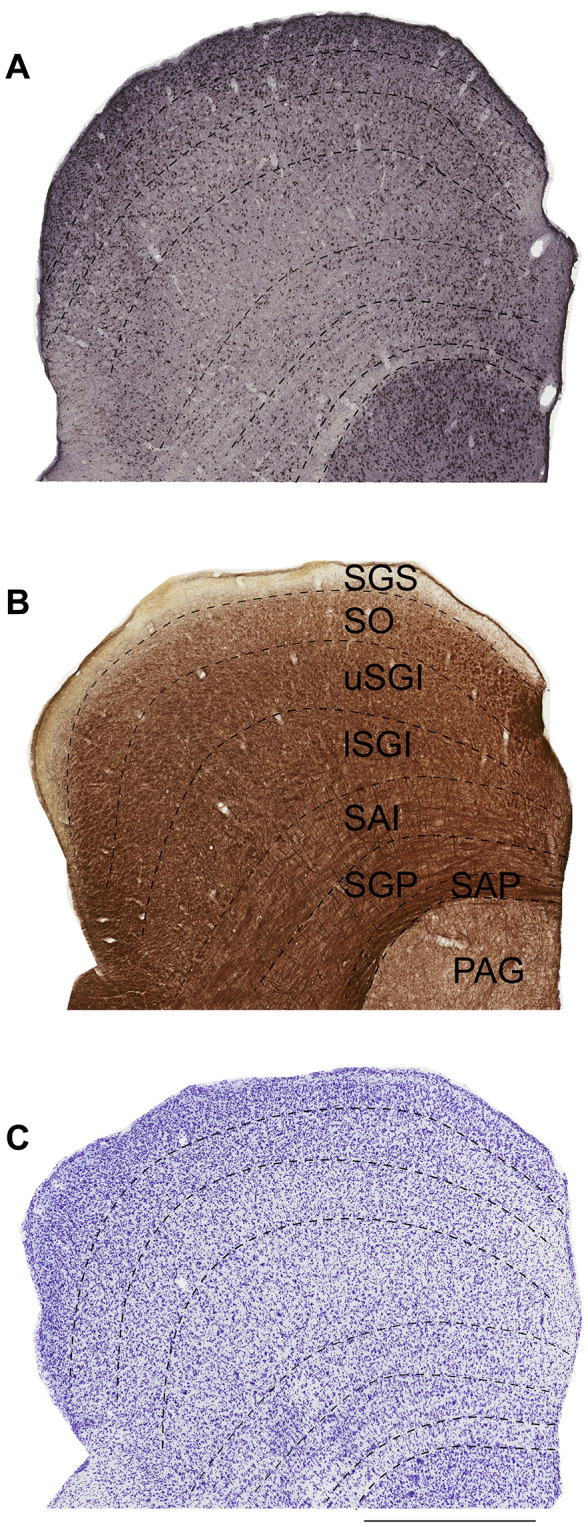
The approximate boundaries of the superior colliculus (SC) layers in the marmoset monkey. Coronal sections stained with NeuN **(A)**, myelin **(B)** and Nissl **(C)** representing interaural levels near the central part of the SC (Paxinos et al., 2012). Dashed lines indicate the boundaries between the layers. Scale bar: 1 mm, SGS, stratum griseum superficiale; SO, stratum opticum; uSGI, upper stratum griseum intermedium; lSGI, lower stratum griseum intermedium; SAI, stratum album intermedium; SGP, stratum griseum profundum; SAP, stratum album profundum; PAG, periaqueductal gray.

An Aperio Scanscope AT Turbo (Leica Biosystems) was used to scan sections stained for SST, VIP and NPY using DAB immunohistochemistry ( × 20 magnification, resolution: 0.5 μm/pixel). Neuronal counts were obtained from at least three coronal SC sections 200 μm apart, approximately from the central part of the SC [including AP level +1.0 mm (Paxinos et al., 2012)]. Similar to our previous report (Chong et al., 2022), we placed counting frames (dimensions: 150 × 100 μm) at equal distances across the mediolateral extent of all SC layers (identified using Nissl, NeuN and myelin staining) except (stratum zonale) SZ and SAP which had no or very few cells (Figure 1). The top and right borders of each counting frame were considered the inclusion lines, and the left and bottom borders the exclusion lines and thus cells within the frame or those touching the inclusion lines were counted (Atapour et al., 2017; Chong et al., 2022). Only neurons with clear nuclei were counted, independent of size and shape.

In calculating the number of neurons per volume unit, both the section thickness (40 μm) and a shrinkage factor of 0.801 (Atapour et al., 2017, 2019) were considered. Average data from consecutive sections taken from middle of the SC were combined to calculate the mean neuronal density of SST+ neurons. The most caudal or rostral sections, which do not include all SC layers, were excluded.

For co-localization analysis, we assessed GABA expression in up to 200 SST+ positive neurons present in different layers/sections using images taken by Nikon C1 laser scanning confocal microscope. All statistical analyses were conducted using GraphPad Prism v10.3.1 (Graph-Pad Software, La Jolla, CA, USA). Simple linear regression or one-way ANOVA with *post*-*hoc* Tukey comparisons test was conducted for statistical comparison.

## Results

The approximate boundaries of the SC layers in the marmoset monkey were determined using complementary information from sections stained with Nissl, myelin and NeuN as described previously (Chong et al., 2022, Figure 1). The most dorsal cellular layer, SGS, showed a densely packed population of neurons in both NeuN and Nissl stain and appeared to be more myelinated toward its lower limit (Figure 1). Within the SO, alternating cell-dense and cell-sparse regions were visible best in the NeuN stain, and the presence of myelinated fiber bundles helped define its limits. Large neurons characterized the SGI, which could be further divided into upper and lower subdivisions based on neuronal density. The transition from SGI to the SAI was marked by a more uniform distribution of smaller neurons. A specific myelin fiber pattern was observed in SAI and SGP. The SAP appeared as a thin, darkly myelinated layer containing few neurons (Figure 1).

### SST+ neurons in the marmoset SC

**Figure 2:**
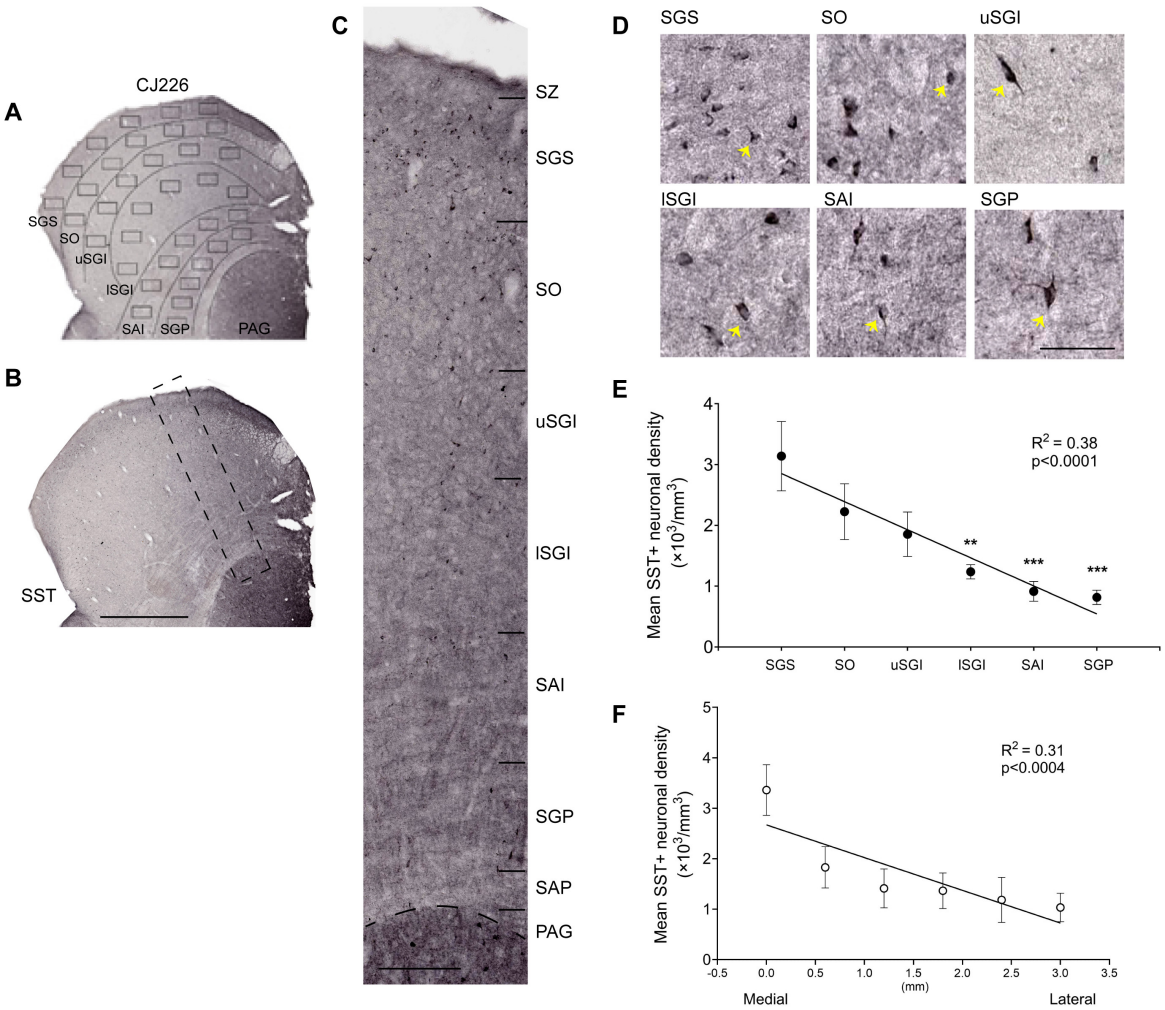
Somatostatin (SST) expression pattern in neurons of superior colliculus (SC) in marmoset monkey. **(A)** Counting frame placement. Lines separate layers with six sampling frames (100 × 150 μm each) placed for each layer as detailed in methods (Chong et al., 2022). **(B)** Representative coronal section of SST-stained SC form animal CJ226. The dashed rectangle is shown in higher magnification in **(C)**. **(D)** SST-positive (SST+) neurons from all layers. Yellow arrow points to some of these neurons. **(E)** SST+ neuronal density across the dorsoventral and **(F)** mediolateral axis of SC. Data shown is mean ± SEM. Statistical comparisons: **p < 0.01, ***p < 0.001 for comparison with SGS. Scale bar: **(A, B)** 1mm, **(C)** 200 μm, **(D)** 50 μm. SC layer abbreviations as per Figure 1.

Immunostaining for SST revealed the presence of SST+ neurons across the SC layers (Figure 2). The cell bodies of SST+ neurons had different shapes, including many elongated cells. Although the full morphologies were not clearly visible by immunostaining, they showed bipolar and multipolar features (Figures 2C, D). The cell bodies measured approximately 6–20 μm at the longest axis and 3–9 μm at the shortest axis. Those observed in the intermediate layers were generally larger in size, consistent with the presence of large neurons in these layers (Bourne and Rosa, 2003; Chong et al., 2022). The mean density of SST+ neurons was estimated to be about 1,698 ± 364.5/mm^3^ based on stereological sampling in 3 cases. The density of SST+ neurons was highest in the superficial layers, creating a gradient across the dorsoventral axis of SC (Simple linear regression; R^2^ = 0.38, *p* < 0.0001), with the SGS having the highest density [Figures 2C, E, SGS: 3,140, SO: 2,230, uSGI: 1,850, lSGI: 1,240, SAI: 910, SGP: 820/mm^3^, One-way ANOVA; F _(5, 48)_ = 2.94, *p* = 0.02, Tukey's multiple comparisons; SGS vs. lSGI: *p* < 0.01 and SGS vs. SAI or SGP: *p* < 0.001]. The SST+ neuronal density was also higher in the medial side of SC, which corresponds to the representation of the upper contralateral quadrant (Chen et al., 2019; Santana et al., 2023), revealing a decreasing mediolateral gradient for average neuronal density across all layers (Figure 2F, Simple linear regression; R^2^ = 0.31, *p* < 0.0004). However, we found a similar distribution of these neurons across the rostrocaudal extent of SC, which roughly corresponds to the gradient of representation from central to peripheral vision (Simple linear regression; R^2^ = 0.06, *p* = 0.20). This contrasts with our previous finding (Chong et al., 2022) of a significant increasing gradient of total neuronal density from rostral to caudal SC.

**Figure 3:**
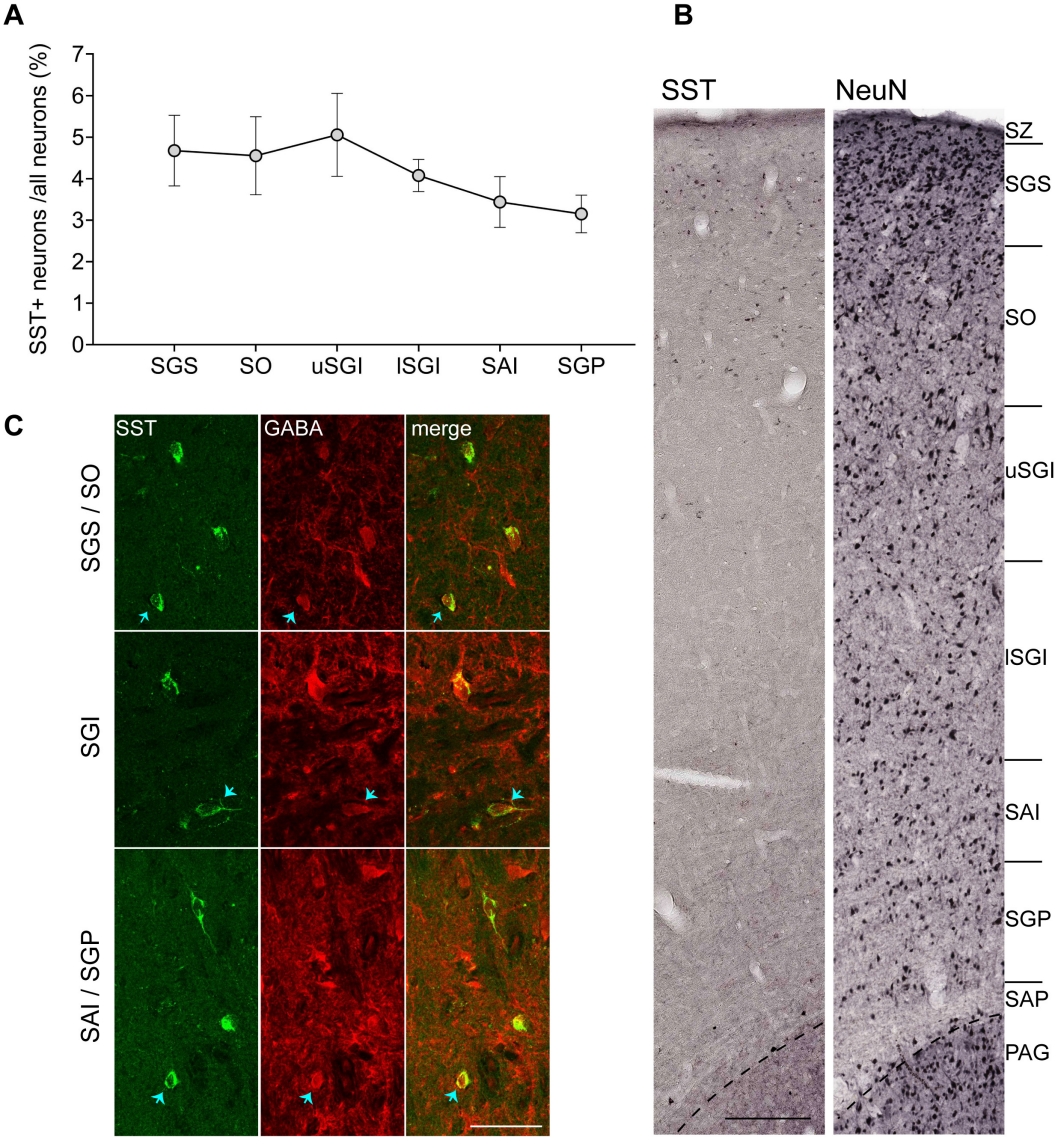
Somatostatin-positive (SST+) neurons are of inhibitory nature and contribute to a small percentage of neurons. **(A**) Proportion of SST+ neurons calculated as percentage all SC neurons stained by NeuN (mean ± SEM, Chong et al., 2022). **(B)** Representative strips covering all SC layers show distribution of SST+ neurons compared to all neurons. **(C)** Representative double immunofluorescence staining for SST and GABA is shown for SGS/SO (top), SGI (middle) and SAI/SGP (bottom) layers obtained from subject CJ249. Arrows point to some of the cells. Scale bar: **(B)** 200 μm, **(C)** 50 μm. SC layer abbreviations as per Figure 1.

SST+ neurons constituted approximately 3 – 5% of total SC neurons, based on estimations of total neuronal density using NeuN staining (Chong et al., 2022). This proportion was relatively similar across layers (Figures 3A, B; SGS: 4.67%, SO: 4.55%, uSGI: 5.05%, lSGI: 4.08%, SAI: 3.44%, SGP: 3.15%).

Double immunofluorescence staining for SST and the neurotransmitter GABA confirmed the inhibitory nature of SST+ neurons (Figure 3C). Our analysis in 200 SST+ neuronal images taken by confocal microscopy revealed that they all co-express GABA. While we cannot exclude the possibility of a small population of excitatory SST+ neurons, it is clear that the SST+ neurons are primarily GABAergic.

### Lack of immunostaining for VIP and NPY in the SC

**Figure 4:**
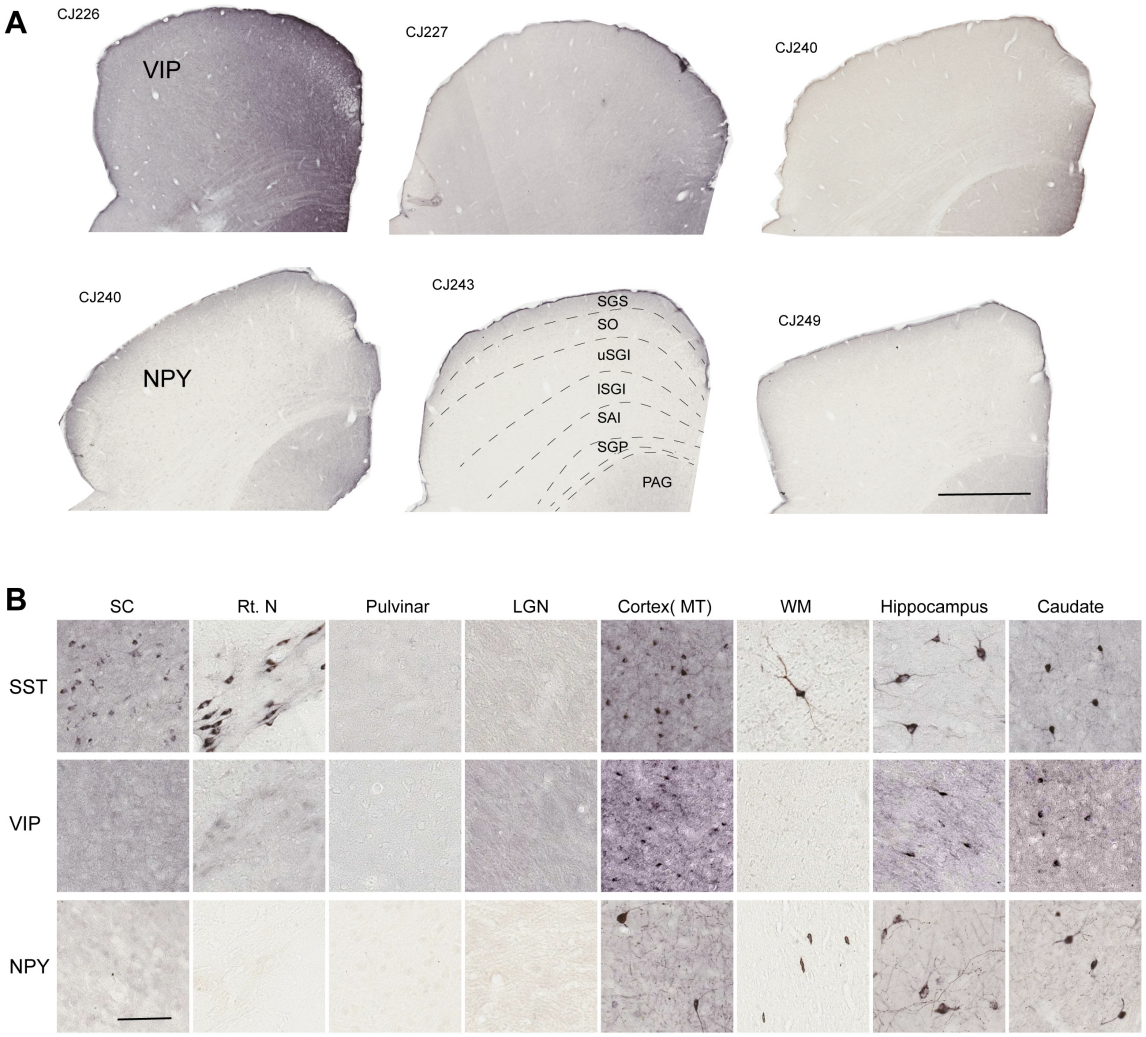
Lack of protein expression for neuropeptide Y (NPY) and vasoactive polypeptide (VIP) in the superior colliculus (SC) neurons of marmoset monkeys. **(A)** Representative immunostained sections approximately from the middle of SC (Paxinos et al., 2012) stained for VIP (top) and NPY (bottom) in three subjects. Subject IDs are shown on the top left side of each section (see Table 1). **(B)** Close-up images of somatostatin-positive (SST+), VIP-positive (VIP+) and NPY-positive (NPY+) neurons present in cortical and subcortical structures in the stained sections. Scale bar: **(A)** 1mm, **(B)** 100 μm. Rt. N, Thalamic reticular nucleus; LGN, lateral geniculate nucleus; MT, Middle temporal area of cortex; WM, white matter. SC layer abbreviations as per Figure 1.

Our results revealed no examples of VIP+ and NPY+ neurons in the SC (Figure 4A) despite the clear presence of neurons stained for both peptides in the cortex and subcortical structures including thalamic reticular nucleus, hippocampus and caudate nucleus, present in the same sections (Figure 4B). This observation was consistent across sections covering the entire SC in each of the three stained cases, suggesting lack of expression of these peptides in the SC of marmoset monkey.

## Discussion

This study confirms that neurons of the SC in the marmoset monkey express SST, but not VIP or NPY adding to the knowledge of SC neurochemistry in primates, where data on the neuronal diversity of the SC remain scarce. SST+ neurons are inhibitory, as suggested by GABA expression, and form a small fraction of neurons in this structure.

### Marmoset SC contain GABAergic SST+ neurons

Identifying neuronal subtypes is crucial for understanding circuitry, connectivity and function (Liu et al., 2023; Sans-Dublanc et al., 2021). SST expression in neurons of marmoset SC is consistent with findings in other mammalian species (Laemle et al., 1982; Harvey et al., 2001; Byun et al., 2016; Spangler and Morley, 1987; Mensah-Brown and Garey, 2006; De Souza et al., 2015). A more prominent SST expression in the superficial layers, confirmed by immunostaining (Harvey et al., 2001; Mensah-Brown and Garey, 2006) and gene expression data (Shimogori et al., 2018), are indicative of a possible role of these neurons in visual processing. Further validating this idea is the observation that, contrary to the higher density of overall neuronal populations in the caudal parts of SC (Chong et al., 2022), the rostrocaudal distribution of SST+ neurons is mostly uniform, suggesting that there may be relatively more SST+ neurons present in the anterior part of marmoset SC, which receives foveal visual input (Chen et al., 2019; Hafed et al., 2021). Moreover, our observations on the expression of SST in thalamic reticular nucleus neurons (Figure 4), along with the known role of these neurons in modulation of visual information processing (Bu et al., 2025) suggest a conserved role of SST+ neurons in visual processing across different mammalian species along with ample evidence of involvement of SST+ neurons of visual cortex to such processes (Rikhye et al., 2021; Song et al., 2020; Ma et al., 2010; Onorato et al., 2025).

Apart from the dorsoventral and rostrocaudal distribution of SST+ neurons discussed above, we also observed a mediolateral gradient for these neurons across SC. However, due to lack of quantitative data in other species (Byun et al., 2016; De Souza et al., 2015; Harvey et al., 2001; Mensah-Brown and Garey, 2006; Spangler and Morley, 1987), the functional implications of this gradient remain to be understood. Data in other species of primate indicate asymmetries in visual processing between the upper and lower visual fields (Jóhannesson et al., 2018; Zito et al., 2016), including roles such as attention (Palmieri et al., 2023).

While inhibitory nature of SST+ neurons in cortex, hippocampus and some other structures are well known (Yavorska and Wehr, 2016), to the best of our knowledge, there has been no study in primates investigating directly whether SST+ neurons in SC were inhibitory. In the cat SC, particularly in the SGS, the SST+ neurons have the morphology of local interneurons (Spangler and Morley, 1987). In the mouse SC, previous *in-situ* hybridization data (Harvey et al., 2001), along with more recent transcriptomic studies (Choi et al., 2023; Liu et al., 2023; Cheung et al., 2024) define SST as a specific GABAergic subtype in the SC. Here our study provides direct evidence for the inhibitory nature of SST+ neurons in the SC of marmoset monkey, while their role in visual processing and perception remains to be tested.

### Lack of VIP and NPY in marmoset SC

We observed an absence of NPY protein expression in the marmoset SC, despite its reported expression in the cat and rodent (Coveñas et al., 1990; Harvey et al., 2001). While NPY gene expression has been reported in the rat SC, mostly within the superficial layers (Harvey et al., 2001), marmoset SC shows very low level of gene expression for NPY only at birth, but not in adulthood (Shimogori et al., 2018; Kita et al., 2021) consistent with our immunohistochemical data. Similar to marmosets, studies in lemurs and squirrel monkeys also reported an absence of NPY+ cell bodies in the SC (Bons et al., 1990; Smith et al., 1985). The absence of NPY in primate SC, along with the increased neocortical NPY innervation, particularly in humans and great apes (Raghanti et al., 2014), may highlight the primate specializations in the circuitry roles involving this neuropeptide. NPY-containing neurons were present in cortex and other subcortical structures such as caudate and hippocampus (Figure 4), where they are known to influence a variety of physiological and cognitive functions (Thorsell et al., 2006).

The absence of VIP protein expression is consistent with lack of its gene expression in the marmoset SC (Shimogori et al., 2018; Kita et al., 2021). Limited data in other species, such as expression of both VIP protein and gene in rodent SC (Byun et al., 2016; Dussaillant et al., 1992; Ogawa-Meguro et al., 1992; Okamoto et al., 1990), vs. its absence in the cat SC (Borostyánkoi et al., 1999), make it clear that there is no uniform expression of these peptides in different mammalian species. Furthermore, it points to obvious differences in the cellular circuitry of the SC in the marmoset, and perhaps other primates, compared to rodents.

SST and VIP immunostaining requires optimal fixation (as specified in the methods section) to label reliably all of the cell populations in different structures. Difficulty of staining could be a contributing factor behind the significant lack of immunostaining data for VIP and SST in the literature. However, in the present study we found clearly labeled neurons in other structures, processed in the same batches and in many cases in the same sections.

Finding out molecular markers that define specific cell types in SC is essential for better understanding of its circuitry, including accurate biophysical models of cellular interactions underlying behavior. Our data point to the neurochemistry of the adult SC neurons in a non-human primate which has become important in studies of vision (Solomon and Rosa, 2014) that might be different during development and aging as well as chronic conditions associated with disease or injury. Future studies should characterize the full morphology of SST+ neurons using other techniques including cell filling and higher-resolution imaging to better understand layer-specific axonal and dendritic distributions and overall SC microcircuit organization.

## Data Availability

The raw data supporting the conclusions of this article will be made available by the authors, without undue reservation.
